# Approaches to Computational Strain Design in the Multiomics Era

**DOI:** 10.3389/fmicb.2019.00597

**Published:** 2019-04-05

**Authors:** Peter C. St. John, Yannick J. Bomble

**Affiliations:** National Renewable Energy Laboratory, Golden, CO, United States

**Keywords:** constraint-based methods, kinetic metabolic models, machine learning, multiomics, strain engineering

## Abstract

Modern omics analyses are able to effectively characterize the genetic, regulatory, and metabolic phenotypes of engineered microbes, yet designing genetic interventions to achieve a desired phenotype remains challenging. With recent developments in genetic engineering techniques, timelines associated with building and testing strain designs have been greatly reduced, allowing for the first time an efficient closed loop iteration between experiment and analysis. However, the scale and complexity associated with multi-omics datasets complicates manual biological reasoning about the mechanisms driving phenotypic changes. Computational techniques therefore form a critical part of the Design-Build-Test-Learn (DBTL) cycle in metabolic engineering. Traditional statistical approaches can reduce the dimensionality of these datasets and identify common motifs among high-performing strains. While successful in many studies, these methods do not take full advantage of known connections between genes, proteins, and metabolic networks. There is therefore a growing interest in model-aided design, in which modeling frameworks from systems biology are used to integrate experimental data and generate effective and non-intuitive design predictions. In this mini-review, we discuss recent progress and challenges in this field. In particular, we compare methods augmenting flux balance analysis with additional constraints from fluxomic, genomic, and metabolomic datasets and methods employing kinetic representations of individual metabolic reactions, and machine learning. We conclude with a discussion of potential future directions for improving strain design predictions in the omics era and remaining experimental and computational hurdles.

## Introduction

The biorefinery concept involves the development of sustainable and low-impact production routes for major commodity chemicals and fuels from biomass (Bozell and Petersen, 2010). Biomanufacturing using engineered microbes is a critical component of many production pathways, and offers the opportunity for high selectivity and yield (Nielsen and Keasling, 2016). However, optimizing microbial metabolism for a given process is time intensive and costly, limiting microbial bioconversions at present to only a few commercially successful compounds (Van Dien, 2013; Chubukov et al., 2016). This difficulty is primarily due to the complex relationship between genotype and phenotype, involving regulation at the metabolic, translational, and transcriptional levels. In recent years, the procedure of strain engineering has been formalized through the Design-Build-Test-Learn (DBTL) cycle, which takes advantage of recent improvements in genetic engineering and high-throughput characterization in the Build and Test stages, respectively, to efficiently screen larger libraries of strain modifications (Liu et al., 2015). The Learn and Design stages use computational techniques to interpret experimental results and suggest further modification targets. The Learn step is perhaps the most weakly developed step of the DBTL cycle, and can take the form of a wide range of computational techniques from statistical analysis to detailed simulations (Nielsen and Keasling, 2016). In this minireview, we discuss recent research in methodology for integrating biological data – particularly in the form of multiomics analyses – into developing new and efficient strain designs. We first review relevant experimental considerations from the Test stage and summarize the types of data available for informing strain designs. We next cover constraint based methods, kinetic simulations, and machine learning approaches, as well as recent studies that have used these methods in strain design. Lastly, we finish by discussing available software implementations and future directions for tackling the Learn step.

## Experimental Inputs

A number of recent reviews have covered the growing usefulness of omics approaches in characterizing cell physiology (Petzold et al., 2015; Nielsen, 2017; Becker and Wittmann, 2018; Yurkovich and Palsson, 2018), and therefore we only briefly cover the relevant data generated in typical strain characterization experiments. Frequently used omics data include transcriptomics, proteomics, metabolomics, and fluxomics, which measure gene expression, protein expression, metabolite concentrations, and intracellular fluxes, respectively. Transcriptomics is typically performed using next-generation sequencing methods that quantify relative differences in RNA expression within a given biological sample (Petzold et al., 2015). Relative comparisons between samples are also possible using statistical techniques (Wagner et al., 2012). Due to the similar physical nature of RNA transcripts, transcriptomics approaches are among the easiest to perform at the genome-scale, but their distance from metabolic networks by several layers of regulation makes direct understanding of metabolic function using these data difficult. Proteomics is one step closer to the determination of metabolic fluxes and uses mass spectrometry to quantify protein expression through the amino acid sequences of digested peptides (Kolker et al., 2006). Similar to transcriptomics, proteomics experiments typically measure relative protein expression within a sample, although statistical and experimental methods for comparing relative protein expression between samples are possible (Petzold et al., 2015). Absolute quantification of protein expression is feasible but more difficult, with a range of accuracies depending on the method used (Arike et al., 2012). While more involved than transcriptomics due to protein’s 3D structure and lack of amplification techniques, proteomic analyses are still able to survey a similar fraction of the protein-coding genome (Haider and Pal, 2013). Metabolomics poses an even greater challenge, as the high turnover of metabolites requires fast quenching and processing of samples (Petzold et al., 2015). As a result, the scope of metabolomic analyses are typically restricted to a smaller fraction of the organism’s metabolism. Similar to transcriptomics and proteomics, metabolite concentrations are typically measured as relative quantities in high-throughput exploratory experiments (Lei et al., 2011). Absolute metabolite quantifications are possible in targeted metabolomic studies using external or isotope-labeled standards. Lastly, fluxomics is concerned with accurately measuring internal fluxes of key metabolic reactions directly using isotopic labeling. While an excellent indicator of metabolic state, fluxomics is performed with less frequency than the previously discussed methods due to its experimental difficulty (Blank, 2016). In addition to careful cell culture and sample processing, fluxomics requires an accurate mathematical model that tracks atom transitions during metabolic reactions (Wiechert, 2001). This mathematical model is used in conjunction with 13C isotope labeling patterns to infer fluxes through each reaction, and as a result, inferred fluxes have typically been restricted to the main reactions in central carbon metabolism. However, extensions of MFA to include genome-scale flux analysis have been proposed (Gopalakrishnan and Maranas, 2015). Some genome-scale MFA methods leverage metabolism’s bow-tie structure to constrain fluxes through peripheral pathways with a high degree of confidence (García Martín et al., 2015; Ando and Garcia Martin, 2018).

Even with access to direct measurements of activity for a wide range cellular machinery components, using these data to enhance metabolic flux for a desired pathway remains challenging. We next discuss Learn techniques that synthesize these vast data sources together with generalized knowledge of biological function.

## Learn Methodology

**FIGURE 1:**
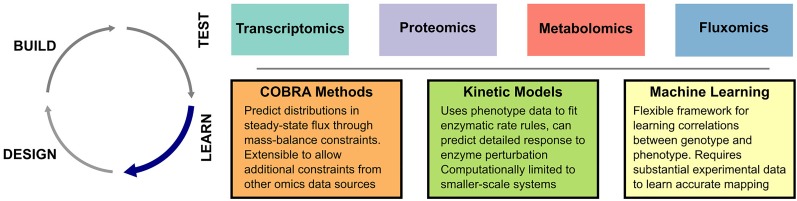
Overview of computational techniques in the Learn step. Omics datasets in Test can be interpreted through a number of different computational strategies.

The goal of the Learn and Design steps is to use the characterization of previously engineered strains to develop improved strain designs. In its most basic form, this step can be accomplished by examining biological features (i.e., differentially expressed genes) correlated with improved strain performance, and overexpressing those likely involved in the pathway of interest (Yoshikawa et al., 2012). Designs based on rational consideration of omics data have proven successful (Guan et al., 2017), validating the human-in-the-loop approach. However, model driven designs will likely be critical to speeding up the DBTL cycle and revealing non-intuitive targets (Vickers, 2016). In the next sections, we review several lines of research into model-driven interpretation of omics data. A schematic of these approaches is shown in Figure 1.

### Constraint-Based Methods

Constraint-Based Reconstruction and Analysis (COBRA) methods use biological knowledge and data to place constraints on intracellular fluxes, and in recent years have expanded to consider a wide range of recent omics techniques. Here we focus on extensions of COBRA methods that pertain to guiding strain designs from omics data, while a number of recent reviews have covered COBRA methods in greater depth (O’Brien et al., 2015; Campbell et al., 2017; Stalidzans et al., 2018). A central technique to COBRA methods is flux balance analysis (FBA), which assumes that metabolite concentrations in the cell reach a pseudo steady-state when compared to the time scales associated with substrate uptake and cell division (Orth et al., 2010). This assumption allows fluxes to be constrained by mass balance equations developed from databases of biochemical reaction stoichiometry. Mass balance constraints alone (in the absence of 13C isotope labeling or other product data) are often not sufficient to determine a unique vector of metabolic fluxes. By assuming a cellular objective such as maximizing biomass or ATP production, unique flux vectors can be predicted. The accuracy of these predicted flux values are dependent on the objective chosen, and some objective functions have shown good correlation with experimental omics data (Lewis et al., 2010). Since such models can be simulated quickly and rely primarily on well-curated databases of metabolic reactions, many genome-scale models (GEMs) of microbial metabolism have been created (Henry et al., 2010; King et al., 2015). While useful in understanding metabolic functionality and predicting the results of gene manipulation, these assumptions are not sufficient to fully incorporate the phenotypic observations resulting from omics analyses.

Extensions to the COBRA framework have therefore been proposed to impose additional constraints from experimental observations. One of the earliest such studies used transcriptomic data to block flux through reactions where gene expression for required enzymes was not observed (Åkesson et al., 2004). This method considered gene product expression through boolean logic, however, more recent studies have explicitly included gene product expression in the constraint-based framework (Becker and Palsson, 2008; Shlomi et al., 2008). Metabolism and gene-expression models (ME-models) explicitly model reactions involved in transcription and translations to build a quantitative model of enzyme production and usage (Lerman et al., 2012). These models therefore allow direct comparison of model predictions with transcriptomic and proteomic data (O’Brien et al., 2014, 2015). In a similar method, genome-scale models with protein structures (GEM-PROs) include structural information about each enzymatically catalyzed reaction (Chang et al., 2013). Such models allow the explicit simulation of the proteome fraction devoted to different cellular activities (Basan et al., 2015), and therefore might also be used to add additional constrains from proteomic analyses. The GECKO method combines literature knowledge of enzyme kinetics with proteomics data to constrain metabolic fluxes (Sánchez et al., 2017). However, while many enzymes have been kinetically characterized for well-studied species, these data are typically not available for non-model microbes (Nilsson et al., 2017).

Metabolomics data are typically incorporated in constraint-based models through the explicit consideration of reaction thermodynamics. If absolute metabolite concentrations are available, thermodynamic metabolic flux analysis can provide more condition-specific information on irreversible reactions (Henry et al., 2007). These principles have been successfully applied to select the most promising pathways for the synthesis of a variety of products (Averesch et al., 2017). Further extensions to the COBRA framework will likely include even more cellular functionality. Toward this goal, whole-cell models that integrate gene expression, protein production, and cell cycle have been constructed (Karr et al., 2012).

Constraint-Based Reconstruction and Analysis methods therefore represent an extensible and computationally efficient framework for connecting omics data of different types and have been used to successfully interpret omics data and improve strain designs in a number of studies (Wisselink et al., 2010; Brunk et al., 2016). An advantage of COBRA methods is their limited number of parameters that must be fit from experimental data, and therefore they are often able to suggest strain designs without substantial experimental support. In particular, these methods are especially efficient in determining metabolic changes that couple product production to cell growth (Long et al., 2015). The accuracy of constraint-based models in predicting *de novo* experimental results has not been rigorously evaluated and would serve a useful study in measuring the progress in our understanding of cellular behavior. However, even modest success rates from predictive tools are useful in guiding experimental efforts where the search space is vast. A limitation of constrained-based methods is that they are often less suitable for suggesting improvements to fine-tune the enzyme expression of an existing pathway. Such a task typically requires a kinetic description of the reactions in question, which we discuss in the next section.

### Kinetic Metabolic Models

The goal of kinetic metabolic models is to capture the dynamic behavior of individual enzymes and integrate these expressions into the behavior of the full metabolic network. These models allow the direct prediction steady-state flux distributions as a function of enzyme expression, which typically serve as the most reliable experimental data for validation. However, models that explicitly incorporate enzyme kinetics (if parameterized correctly) are capable of predicting finer details of pathway dynamics, including the effect of slight changes in enzyme activity on metabolic flux. In constraint-based models, metabolite pools are assumed to be in a pseudo steady-state, and thus the rate rules governing flux through each reaction can be ignored. While the steady-state assumption may be justified, the specific steady-state reached inside the cell is determined, among a multitude of factors, both by external metabolite conditions as well as the kinetics and expression levels of metabolic enzymes. Kinetic modeling frameworks therefore seek to estimate these reaction rate rules from observed metabolic phenotypes to predict how enzyme perturbation will affect steady-state concentrations and fluxes.

Small-scale kinetic models of core carbon metabolism can leverage enzyme kinetics *in vitro* and time-course metabolite concentration measurements in fitting parameter values (Chassagnole et al., 2002). However, transient cellular responses are difficult to measure at the genome-scale, and direct enzyme kinetic measurements are sparser for peripheral pathways. Large-scale dynamic metabolic simulations are therefore largely based on steady-state flux and concentration data (Vasilakou et al., 2016). Because of these limited data, quantifying parameter uncertainty is therefore a critical challenge in large-scale kinetic models (Tummler and Klipp, 2018). Metabolic ensemble modeling addresses this challenge directly by finding distributions in parameter values that all reproduce the observed experimental data (Tran et al., 2008). This approach has been used to suggest subsequent enzymes in a linear pathway for overexpression (Contador et al., 2009), and an ensemble-based kinetic model of *Escherichia coli* has demonstrated superior predictive ability of steady-state flux distributions (Khodayari et al., 2014).

Smaller-scale, hand-curated kinetic models can use rate rules for individual enzymes with experimentally validated functional forms. However, traditional rate rule expressions (such as Michaelis–Menten kinetics) become difficult to construct for reactions with many participating species. Accordingly, larger-scale kinetic models typically choose a generalizable framework for constructing rate rule expressions. These frameworks range in computational complexity and faithfulness to the underlying enzyme-substrate system, and we leave a detailed comparison of these approaches to a number of recently published reviews (Heijnen, 2005; Hadlich et al., 2009; Du et al., 2016; Saa and Nielsen, 2017). Software available for kinetic modeling has continued to improve, and typically allows the user to specify reaction stoichiometry and rate rules independently from the chosen simulation algorithm. Such software includes COPASI (Hoops et al., 2006), CellDesigner (Funahashi et al., 2008), and MATCONT (Dhooge et al., 2003).

Regardless of the framework chosen, a major hurdle in using kinetic models for interpretation of omics data is the computational effort required in parameter estimation. In metabolic ensemble modeling, parameters are sampled at random and retained in the final ensemble only if they match all the considered experimental data (Tran et al., 2008). As a result, as more data is added or the model expanded, the computational costs increase substantially. Methods for improving the computational speed of the approach have been developed (Greene et al., 2017), but calculating steady states of the dynamic model remains a computational bottleneck. Ensemble-based inference approaches are therefore typically applied to smaller, core-carbon metabolic networks (Khodayari et al., 2014). A recent genome-scale kinetic modeling study optimized only a single parameter set due to the added cost of ensemble-based parameter estimation (Khodayari and Maranas, 2016). However, this single parameter set demonstrated a superior ability to reproduce a wide range of experimental observations compared with constraint-based methods (Khodayari and Maranas, 2016). The ensemble modeling sampling approach has been recently formalized as a form of Bayesian inference (Saa and Nielsen, 2016), demonstrating that detailed posterior distributions in parameter estimates and model predictions could be found. Kinetic models therefore offer a promising future direction for incorporating vast quantities of omics data in metabolic reconstructions if computational bottlenecks can be circumvented (St. John et al., 2018). While difficult to fit, the added parameters from kinetic representations give these models more expressive power in fitting experimental data.

A factor complicating the analysis of experimental data with kinetic models is the stochasticity introduced by low cell volumes and small copy numbers of several key enzymes (Levine and Hwa, 2007; Kiviet et al., 2014). Cell to cell heterogeneity therefore imposes unique challenges in understanding microbial kinetics that might be resolved through the use of explicit stochastic simulation algorithms (Gillespie, 1977) as implemented in a variety of software packages (Hoops et al., 2006; Sanft et al., 2011; Abel et al., 2016) In the subsequent section we discuss machine learning approaches that add even more parameters to be fit, but may prove useful as high-throughput strain construction and characterization techniques improve.

### Machine Learning

Machine learning methods for interpreting omics data have taken a wide range of forms, largely due to the many varied biological questions that can be asked. In this section, we focus on methods that predict future targets for strain engineering. Integrative omics analyses attempt to draw connects between disparate omics data sources, either with or without prior biological knowledge (Berger et al., 2013; Bersanelli et al., 2016). These methods have been used to predict key regulatory genes correlated with metabolic productivity (Larsen et al., 2018), and inferred regulatory networks have also been incorporated into FBA models (Chandrasekaran and Price, 2010). Other studies have used machine learning to understand and predict metabolic performance from hyperparameters associated with cell growth. Wu et al. (2016) explored methods for machine learning in meta-analysis to predict likely pathway success as a function of the complexity of the engineered pathway and other factors. In Kim et al. (2016), machine learning methods are used both for data reconciliation between omics sources, as well as to directly map the genotype-phenotype relationship. Another interesting study used machine learning methods as a replacement for the traditional rate equation frameworks discussed in the previous section (Costello and Martin, 2018). In that study, rate equations were learned directly from time-series metabolomics and were successful in predicting medium-producing strains given high and low-producing varieties. Costello and Martin (2018) also quantified the amount of data required for accurate rate determination at approximately 10 strains. Given the rapid advancement of machine learning methods and biological data collection, these approaches may offer flexible and efficient ways of directly incorporate biological data in new strain designs.

## Discussion

Since Learn lags behind the rest of the DBTL methodology in the development of validated and standardized techniques, feasible computational techniques are still being explored and improved upon. As a result, software libraries for performing the analyses described in this minireview are relatively scarce. As the most mature method of the three, COBRA methods have relatively strong software support in both the MATLAB (Heirendt et al., 2017) and Python (Ebrahim et al., 2013) ecosystems. Dependent packages have also been created for a number of the COBRA extensions for integrating or predicting omics-level data. Kinetic models, alternatively, have relatively poor support in the software landscape. This is likely due to the multitude of kinetic frameworks available as well as their slow (but parallelize-able) convergence, requiring hardware-dependent simulation strategies. For machine learning, several actively developed packages are available that implement common approaches. Scikit-Learn for Python implements a variety of machine learning strategies under a consistent API (Pedregosa et al., 2011). Deep learning frameworks such as Tensorflow or PyTorch simplify the process of constructing deep neural networks and training them on specialized hardware. Compared to the availability of general-purpose machine learning, omics-specific machine learning analyses have substantially fewer libraries under active development. However, creating and distributing standardized Learn work flows will be critical to enabling the reproducible analyses required of the iterative DBTL cycle. Such standardized approaches will necessarily require the development and maintenance of software and best practices in the metabolic modeling community.

## Author Contributions

PSJ and YB contributed to the conception and writing of the manuscript. PSJ created the figure. YB supervised the research.

## Conflict of Interest Statement

The authors declare that the research was conducted in the absence of any commercial or financial relationships that could be construed as a potential conflict of interest.
